# Real-world retrospective study on allergy screening and its effect on specialist referral patterns

**DOI:** 10.3389/falgy.2026.1744703

**Published:** 2026-05-04

**Authors:** Inês Costa Farinha, Milagros Lázaro-Sastre, Rosita Castillo Loja, Adelaida Cabrera, Nerea Otero-Fernández, Elena Laffond Yges, Sonia Arriba-Méndez, Ignacio Dávila

**Affiliations:** 1 Allergy and Clinical Immunology Department, Local Health Unit of Coimbra, Coimbra, Portugal; 2 Allergy Department, University Hospital of Salamanca, Salamanca, Spain; 3 Institute for Biomedical Research of Salamanca (IBSAL), Salamanca, Spain; 4 Department of Biomedical and Diagnostic Sciences, University of Salamanca, Salamanca, Spain; 5 Inflammatory Diseases Network (REI). Institute of Health Carlos III, Madrid, Spain

**Keywords:** allergy referral, Phadiatop test, real-world, screening test, specific igE

## Abstract

**Introduction and Objectives:**

Accurate diagnosis of IgE-mediated diseases requires identifying relevant allergens to guide appropriate clinical management and reduce healthcare costs (1). The ImmunoCAP Phadiatop (Thermo Fisher Scientific) is an *in vitro* assay detecting that detects IgE antibodies against a balanced mixture of common inhalant allergens, serving as a first-level screening tool for sensitization in patients with suspected airway allergies, and guiding subsequent management. This study aimed to evaluate test results, the impact on clinical decision-making, and its role in managing allergic conditions.

**Materials and Methods:**

A real-world retrospective observational study was conducted in a tertiary hospital allergy department (June 2021–June 2023). Data were collected using the Modulab Laboratory Information System (Werfen, Spain) and stored in a dissociated database. The Ethics Committee of Salamanca approved the study protocol. Phadiatop values above 0.35 PAU/l were considered positive. In case of repeated samples, the first was selected. Variables analyzed included test results, patient demographics, requesting departments, and allergy referrals, a well-balanced mixture of common inhalant allergens with regional adaptation. The allergen groups included were comprise: house dust mites, (e.g., Dermatophagoides pteronyssinus, D. farinae), pollens, from grasses (e.g., timothy, ryegrass), trees (e.g., birch, olive), and weeds (e.g., mugwort, plantain, ragweed), molds (e.g., Alternaria, Cladosporium, Aspergillus, Penicillium), and animal dander (e.g., cat, dog).

**Results:**

The study included 596 patients (median age 46, 64.1% female). Of these, 12.6% were under 18 years old. Most patients (95.8%) were referred from primary care, with 83.1% of them from urban areas. A total of 37.9% of patients had a positive result (median IgE 7.81 PAU/l). Most positive patients resided in urban areas (82.3%), were aged <18 years of age (*p* = 0.001), and were male (*p* < 0.001). Patients aged >59 (26.3%) presented mainly negative results (82.2%). Of the positive cases, 31.4% were referred to an allergist, with higher Phadiatop values increasing associated with increased referrals (*p* = 0.001).

**Conclusions:**

Phadiatop was mainly utilized in primary care. One-third of patients tested positive; of those, only one-third were referred to allergists, with higher Phadiatop values increasing associated with higher referral rates. Positivity was higher in males and younger people.

## Introduction and objectives

Allergic diseases are a common reason for general consultation. This fact is associated with the prevalence of allergic diseases, which have notably increased worldwide, especially in developing countries (1, 2). In most healthcare systems, the primary care setting is the initial contact for patients seeking medical advice regarding their allergic diseases (3).

Correct diagnosis of immunoglobulin (Ig)E-mediated diseases by identifying offending allergens is a prerequisite for appropriate management by general practitioners (3, 4). Given the significant workload in primary care, the EAACI Task Force for Allergy Management in Primary Care has critically reviewed diagnostic tests and provided simplified pathways for recognizing, diagnosing, and managing the most common allergic diseases seen and treated in primary care. The ultimate goal is to enhance the ability of primary care providers to refer patients with allergies more efficiently (3, 5, 6).

Additionally, avoiding exposure to common allergens is crucial to effectively managing allergic diseases, minimizing the need for medication, and reducing healthcare costs (7). Selecting appropriate testing methods can be challenging, particularly in primary care, where most allergic patients are often first detected (8).

In clinical practice, evidence of allergic sensitization can be elucidated by two methods, namely skin prick tests (SPT) and specific serum IgE assays. In conjunction with a suitable medical history, those results can assist in confirming or refuting specific allergen triggers in an individual. Among the screening methods typically used to identify individuals with allergic disease, SPT is the most established and is regarded as reliable, cost-effective, straightforward, and time-efficient (3, 9). Nevertheless, SPTs require training, are susceptible to individual interpretation, and though the risk of anaphylaxis is extremely remote, it cannot be entirely excluded (10). Screening for aeroallergen sensitization using allergen-specific IgE measurement in serum has become an increasingly common tool for the diagnosis of allergy, with advantages of increased convenience, accessible performance and interpretation, and not requiring discontinuation of medications susceptible to affecting SPT results (3, 9, 11). A disadvantage of serum-specific IgE assays is their higher cost, particularly for multiplex allergen tests. However, these methods are primarily recommended for cases involving high-complexity sensitization profiles or idiopathic anaphylaxis (12).

A multi-allergen IgE screening test has been developed that is more cost-effective and requires only a small amount of serum or plasma (1). ImmunoCAP Phadiatop (Thermo Fisher Scientific, Uppsala, Sweden) is a commercially available semiquantitative variant of a serum-specific IgE assay designed to detect IgE antibodies against a balanced mixture of common allergens that cause airway allergies in both pediatric and adult populations (9, 13, 14). A positive result provides a summarized picture of allergic sensitization, although further discrimination is required, as the first-level test design does not focus on which specific allergens the patient is sensitized to, but rather on their atopy status. Given the complexity of allergy symptoms and the numerous potential confounding factors, and considering that history and physical examination tend to overestimate allergic sensitization, the test enables healthcare providers to identify atopic individuals with purported sensitization to common inhalant allergens. It helps guide subsequent orientation and management. The Phadiatop mix comprises representative inhalant allergens (house dust mites, pollen from grasses, trees, and weeds, molds, and animal dander), with regional adaptation by the manufacturer. However, the exact composition is not specified. These characteristics are specified by the manufacturer for clinical use as an atopy screen.

Multiple studies have reported favorable performance of the Phadiatop test for screening sensitization to a wide range of common inhalant allergens, as well as its cost-effectiveness and efficiency, making it an appropriate first-line screening tool for individuals suspected of having atopic diseases (1, 4, 8, 9, 11, 13–17). It is important to note that Phadiatop is a qualitative screening test and should not be considered the definitive step in allergic diagnosis. When the test is positive, referral to an allergist is highly recommended for an accurate, in-depth evaluation, as Phadiatop is not designed to indicate the specific sensitizing allergen (1).

There is currently no research evaluating the effectiveness of Phadiatop in allergy referrals the primary objective was to evaluate Phadiatop test results and their association with subsequent referral to an allergist and secondary objectives were to analyze demographic factors associated with Phadiatop positivity, and to describe final diagnoses and sensitization profiles in pediatric patients over 2 years and adult patients, with a particular focus on the test's influence on referral patterns.

## Materials and methods

### Study design and setting

A retrospective observational real-world study that evaluated Phadiatop test results was conducted in the Allergy Department of a tertiary hospital over two years, from June 2021 to June 2023. This department is the sole allergy unit within its healthcare area. Data were collected using the Modulab Laboratory Information System (Werfen, Hospitalet de Llobregat, Spain) and stored in a dissociated database. For patients with multiple Phadiatop tests, only the result was considered for analysis. The study included the Phadiatop test results, the medical departments requesting the tests (primary care, secondary care/specialized departments, emergency department), patient demographics (gender, age), the environment where patients lived (urban, semi-urban, or rural), referrals to the allergy department and time-to-referral. Phadiatop values above 0.35 PAU/l were considered positive. In patients referred to specialized allergy care, the final diagnosis was reached through a comprehensive review of the clinical records, together with sIgE testing. After the allergic study, patients were classified as monosensitized if they had a positive result with one group of aeroallergens without cross-reactivity among the groups (e.g., pollens, house dust mites, epithelia, and molds). Patients were classified as polysensitized if they were sensitized to two or more of these groups. The study was prospectively structured around one primary and two secondary objectives to ensure alignment between aims, methods, and reporting. The primary objective was to evaluate Phadiatop test results and their association with subsequent referral to an allergist. Secondary objectives were to analyze demographic correlates of Phadiatop positivity and to describe final diagnoses and sensitization patterns among referred patients.

Age categories were standardized across the study as <18 years, 18–59 years, and >59 years. Classification of patients’ residence as urban, semi-urban, or rural was based on the administrative geographic categories assigned in the health area at the time the Phadiatop test was requested.

In our health area, Phadiatop is typically ordered by primary-care physicians when patients present with rhinitis, conjunctivitis, or asthma symptoms suggestive of aeroallergen sensitization. However, this is a real-world study, and during the study period, no unified protocol guided test ordering, and clinical details such as symptom severity or seasonality were not systematically recorded, which could be a limitation We are unaware of evidence-based guidelines recommending referral solely based on Phadiatop results

We defined four inhalant groups: mites (Dermatophagoides pteronyssinus, D. farinae), pollens (grasses, trees, weeds), molds (Alternaria, Cladosporium, Aspergillus, Penicillium), and animal dander. These categories reflect standard clinical practice and including the relevant aeroallergens prevalent in our area.

SPT was performed with standardized aeroallergen extracts according to routine clinical protocols. The complete SPT panel used at our center will be detailed in Table 1.

**Table 1:** Allergens used for skin prick testing.

Allergen Group	Common Name	Scientific/Latin Name
Mites	House dust mite	*Dermatophagoides pteronyssinus*
	House dust mite	*Dermatophagoides farinae*
	Storage mite	*Acarus siro*
	Storage mite	*Tyrophagus putrescentiae*
	Storage mite	*Lepidoglyphus destructor*
Fungi (Molds)	Alternaria	*Alternaria alternata*
	Aspergillus	*Aspergillus fumigatus*
	Cladosporium	*Cladosporium herbarum*
	Penicillium	*Penicillium chrysogenum*
Grasses, Weeds	Bermuda grass	*Cynodon dactylon*
	Timothy grass	*Phleum pratense*
	Mugwort	*Artemisia vulgaris*
	Goosefoot	*Chenopodium album*
	Parietaria	*Parietaria judaica*
	Plantain	*Plantago lanceolata*
Tree Pollens	Cypress	*Cupressus sempervirens*
	Olive tree	*Olea europaea*
	Plane tree	*Platanus acerifolia*
	Oak	*Quercus robur*
	Birch	*Betula pendula/Betula verrucosa*
Animal Dander	Horse	*Equus caballus*
	Rabbit	*Oryctolagus cuniculus*
	Cat	*Felis catus*
	Hamster	*Mesocricetus auratus*
	Dog	*Canis familiaris*

sIgE determinations using the ImmunoCAP platform (Thermo Fisher Scientific) were obtained from the patientśclinical records when available.

### Statistical analysis

The distribution of continuous variables was assessed for normality. Non-normal variables were summarized as the median and interquartile range (IQR, 25th–75th percentile). Categorical variables were described using absolute and relative frequencies. Group comparisons were performed using the Mann–Whitney U test for continuous variables and the chi-square or Fisher's exact test for categorical variables. Statistical analyses were performed using IBM SPSS Statistics v29 (IBM Corp., Armonk, NY).

Variables showing statistically significant differences in bivariate analyses (*p* < 0.05), specifically age, sex, and any other variable meeting this threshold, were considered candidate predictors for logistic regression. These were first evaluated through univariate models and then entered into a multivariable logistic regression model. Potential multicollinearity was assessed by examining correlations, and no problematic multicollinearity was detected. Model fit was evaluated using the goodness-of- statistics provided by the software, which indicated an acceptable fit for the final model. A p-value < 0.05 was considered statistically significant.

### Ethical considerations

The study conformed to the principles of the Declaration of Helsinki. It was approved by the Ethics Committee of the Health Area of Salamanca (PI/2023/12/1465), ensuring adherence to ethical research standards.

The study was funded by a research grant provided by Thermo Fisher Scientific. Grant ID: CABAL2403.

The funder had no role in study design, data access, data extraction, statistical analysis, interpretation of results, or the decision to submit this manuscript. All data handling and analyses were performed independently by the authors, who retained full control over the study.

## Results

Five hundred ninety-six patients were included in the study, predominantly female (64.1%). The median age of participants was 46 years (IQR 25%–75%, 26–61), with a minimum age of 2 years and a maximum of 103 years. The age distribution of patients was as follows: 75 patients were under 18 (12.6%), and 157 were over 59 (26.3%). Primary care physicians ordered most tests (95.8%), and 83.1% of patients lived in urban areas.

### High incidence of negative results observed

Concerning Phadiatop results, 37.9% (*n* = 226) were positive. The median value of positive tests was 7.81 PAU/l (IQR 25%–75% 1.8 −24.5). Positive test results were more frequently observed in patients residing in urban areas (82.3%; *p* = 0.327), in patients under 18 years old (*p* = 0.001), and in male patients (44.2% vs. 30.8%, *p* < 0.001). A higher prevalence of negative Phadiatop results was observed in patients over 59 years (*n* = 157; 82.2% had a negative result). A statistically significant age difference was observed between patients with positive and negative Phadiatop results [[mean ages 33 vs. 52 years, respectively, *p* < 0.001, OR = 0.97 (CI = 0.95–0.99)] (Table 2). After logistic regression, only younger age (OR = 0.97, *p* < 0.001) was identified as an independent factor for a positive test result (Table 3).

**Table 2:** Comparison of the baseline demographic characteristics of patients with positive or negative phadiatop assay results.

Variables, *n* (%)		Total 596 (100)	Phadiatop		*p*-value
			Positive 226 (37.9)	Negative 370 (62.1)	
**Sex**	Female	382 (64.1)	126 (55.8)	256 (69.2)	**<0**.**001**^¥^
	Male	214 (35.9)	100 (44.2)	114 (30.8)	
**Age** (years)^Ø^		46 (26–61)	33 (21–47)	52 (33–67)	**<0.001** ^#^
**Requesting departments**	Primary care	571 (95.8)	215 (95.1)	356 (96.2)	0.431^§^
	Secondary care	24 (4.0)	10 (4.4)	14 (3.8)	
	Emergency	1 (0.2)	1 (0.4)	0 (0)	
**Environment**	Urban	495 (83.1)	186 (82.3)	309 (83.5)	0.617^¥^
	Suburban	33 (5.5)	11 (4.9)	22 (5.9)	
	Rural	68 (11.4)	29 (12.8)	39 (10.5)	

Ø median (interquartile range).

# non-parametric Mann–Whitney U test.

¥ Chi-square test (*χ*2).

§ Fisheŕs exact test.

Continuous variables summarized as median (IQR); categorical as *n* (%).

Continuous variables were analyzed using the Mann–Whitney U test, and categorical variables were compared using the chi-square test or Fisher's exact test, as appropriate.Phadiatop values above 0.35 PAU/l were considered positive. *p* < 0.05 represents statistically significant (marked with bold).

**Table 3:** Univariate and multivariate logistic regression analysis of predictive factors for a positive phadiatop result.

Variable	Univariate		Multivariate	
	*OR* (95% - CI)	*p*-value	*OR* (95% - CI)	*p*-value
**Age (years)**	0.97 (0.95–0.99)	**<0.001**	0.97 (0.95–0.99)	**<0**.**001**
**Male gender**	1.13 (0.64–1.99)	0.683	1.37 (0.76–2.49)	0.296
95% - CI, 95% confidence interval; *OR*, *Odds Ratio*.				

*p* < 0.05 represents statistically significant (marked with bold).

### Referral of patients with a positive result to an allergist was infrequent and delayed

**Figure 1:**
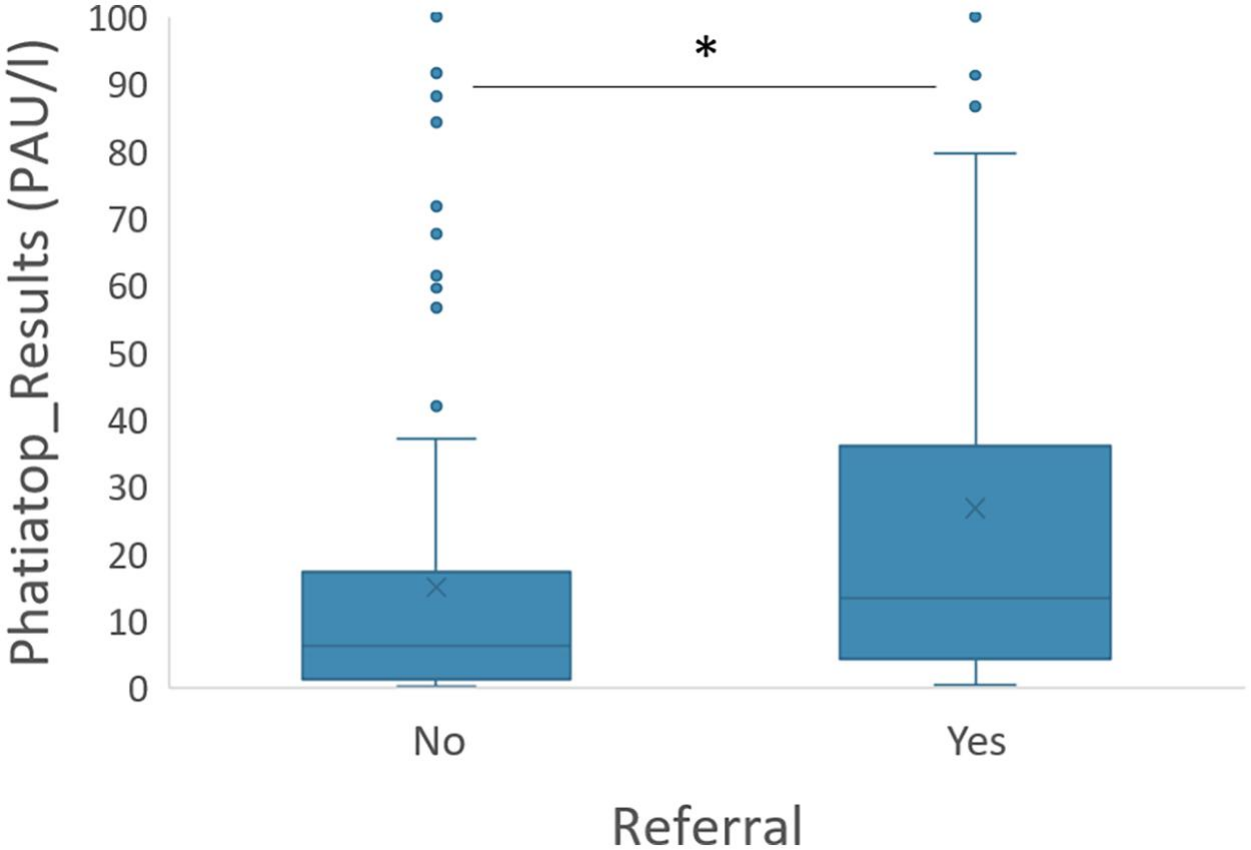
Distribution of phadiatop values among patients referred to the allergy department compared to those not referred [non-parametric Mann–Whitney U test (* indicates *p* = 0.001)].

Only 71 out of 226 patients with a positive result (31.4%) were referred to an allergist. There was a significant increase in the number of referrals when Phadiatop values were higher (*p* = 0.001) (Figure 1). The median duration from the primary care physician receiving the result to the visit to the allergy specialist was 116 days (IQR 25%–75%: 57–210 days).

**Figure 2:**
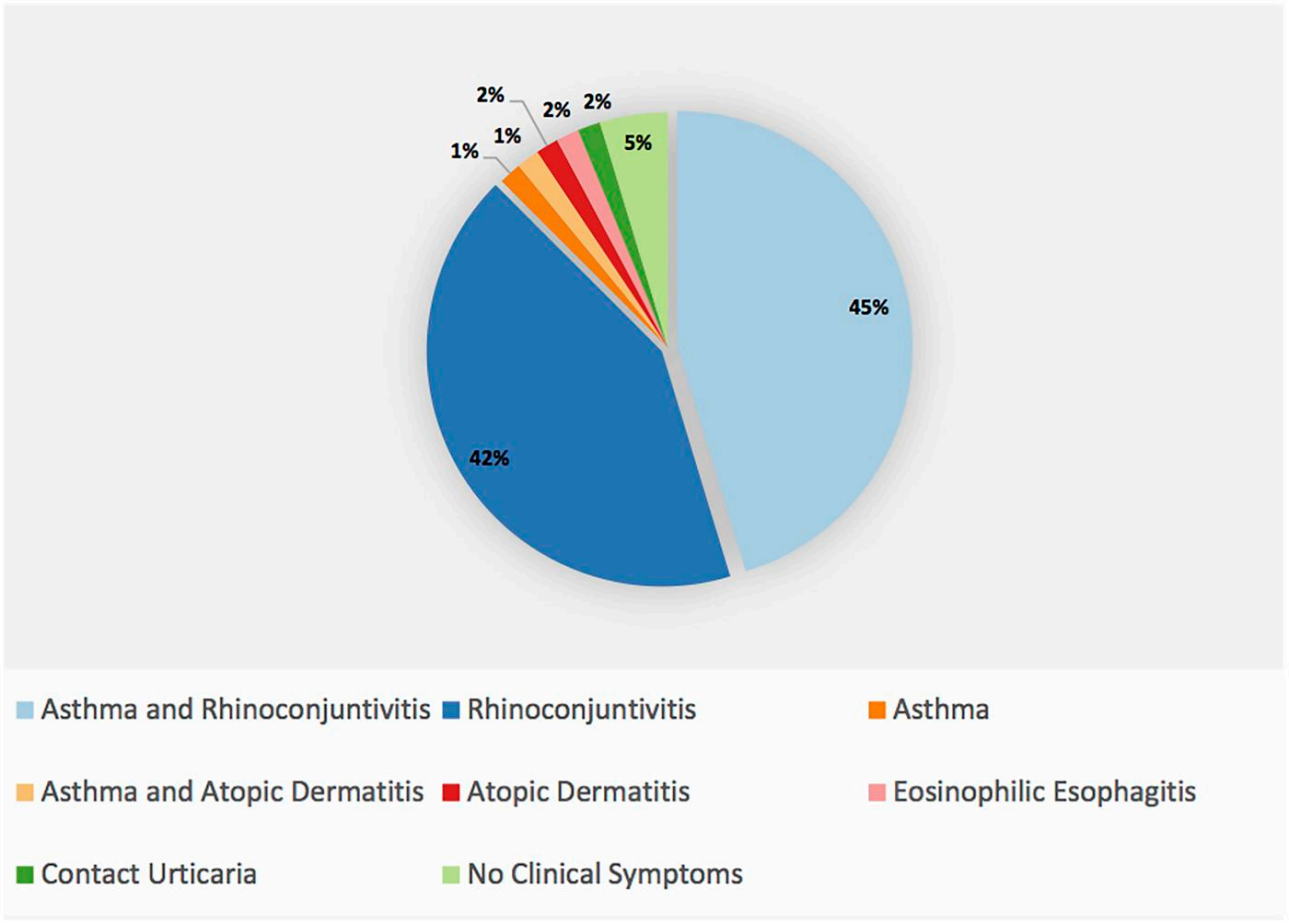
Final allergist-established clinical diagnoses (percentage).

Following referral (Figure 2), 29 patients were diagnosed with both asthma and rhinoconjunctivitis, and 27 with rhinoconjunctivitis alone. Other diagnoses included asthma (1 case), asthma with atopic dermatitis (1 case), eosinophilic esophagitis (1 case), and contact urticaria (1 case). Notably, three patients showed no clinical symptoms despite having a positive Phadiatop test result.

### Referred patients were polysensitized

In our evaluation of allergic sensitization in referred patients using SPT and specific IgE testing, we found that 18 patients were sensitized to house dust mites, 6 of whom were monosensitized. Additionally, 54 patients were sensitized to pollen, 24 of whom were monosensitized. Six patients were sensitized to fungi, none of whom were monosensitized, and 24 patients were sensitized to epithelia, with one being monosensitized. Furthermore, two patients had negative skin prick tests, and eight did not undergo testing due to missed appointments or lack of clinical indication. Overall, 31 patients, which represents 43.7% of the referred patients, were monosensitized.

## Discussion

Phadiatop was mainly used in primary care settings, as expected, which is consistent with its intended design and purpose. One-third of patients had a positive result, a proportion that was higher among patients <18. Remarkably, only one-third of patients with a positive result were referred to allergists, with referral rates increasing with higher Phadiatop values.

The fact that most of the patients were referred from primary care indicates that the test is accessible and considered appropriate by the primary health care practitioners’ community for use as a frontline screening tool for allergic sensitization. This aligns with the recommended use, given its high sensitivity and specificity in identifying individuals sensitized to common inhalants (8). Moreover, the fact that 70% of tested patients had negative results un,derscores the test's usefulness as it helps confirm or rule out sensitization, thereby avoiding the prescription of inappropriate treatments.

We observed age-related variability in test results, with younger patients showing higher rates of positivity, consistent with existing literature on the epidemiology of allergic diseases, which indicates that airway allergic conditions are more common in childhood, with sensitization to inhalant allergens developing at an earlier stage (18). It is noteworthy that positive results were observed in only one-third of patients. Older patients were less likely to exhibit positive results, which may reflect a decline in allergic sensitization with age.

Although the odds ratio for age (OR = 0.97) was statistically significant, the effect size was small, indicating only a minimal change in the odds of the outcome per additional year. Therefore, its clinical impact should be interpreted cautiously; larger prospective samples may clarify the magnitude and relevance of this association.

Overall, results show that the criteria for Phadiatop prescription were not as appropriate as they should have been. Additionally, only 12.6% of the sample population was under 18, the age group with the highest frequency of positive results. That suggests that, in the pediatric population, the test is underused and that educational efforts should also be directed at primary care pediatricians. Our results are consistent with EAACI's recent study on the confidence of Primary Care Pediatricians in diagnosing, managing, and referring patients with allergies, which demonstrated knowledge gaps and educational needs in allergy clinical practice (18). This finding is consistent with the cohort's age distribution (older patients showed a higher prevalence of negative results) and with the likelihood that many nasal, conjunctival, or bronchial symptoms assessed in primary care have non-allergic etiologies. We therefore avoid attributing causality to any misunderstanding of test methodology.

The one-third referral rate observed among patients with a positive test result is relatively low and suggests a potential knowledge gap about the utility of this test and the need for accurate allergy diagnosis by allergy specialists. As Phadiatop is a screening tool that should not be considered as the definitive step in assessing allergic conditions (1). Further education among primary care physicians is needed regarding the appropriate use of the Phadiatop test and the importance of referring patients after a positive screening to ensure optimal management of allergic conditions. Moreover, educating patients on the importance of accurate allergy diagnosis is also essential.

Despite extensive research, we could not identify studies with a comparable design to ours. In Spain, Vidal et al. (9) evaluated the diagnostic accuracy of Phadiatop for the detection of allergic sensitization in a general adult population referred from secondary care and selected by an invitation letter, concluding that Phadiatop was a valuable tool for the diagnosis of allergic sensitization in a general adult population, with better specificity than sensitivity. It should be noted that other studies have also analyzed Phadiatop test performance in patients with asthma or rhinitis (11, 12). However, none of the peer-reviewed studies mentioned can be directly compared with our study's results due to differences in design and objectives. In addition, because our study was conducted under real-world conditions, its results may more accurately reflect the test's application in practice.

Interestingly, higher Phadiatop values were significantly associated with increased referrals. Despite Phadiatop being developed by the manufacturer as a qualitative test to support the diagnosis of atopy, data from this study and others suggest that physicians are basing their decisions on the numerical value of the test (PAU/l), possibly linking it to a higher sensitization load (14, 16). This again highlights a gap in knowledge about the test's intended purpose and appropriate use, despite its having been commercially available for almost 20 years.

Finally, the median time from referral to an Allergy specialist consultation was about four months. Notwithstanding, it should be noted that not all patients were immediately referred after a positive diagnosis. Regarding the potential influence of waiting lists on referral patterns, the usual waiting time for an allergy appointment in our system is around three months and is relatively stable. Therefore, we believe that waiting-list duration was not a major factor contributing to the low referral rate. Once more, no similar studies have been identified for comparison.

The study's main limitation is its retrospective design, which may be associated with a lack of variables in some patients, but, in general, most data were obtained. This could be attributed to the study being a monocenter study, which allows for greater data uniformity. Patient age distribution is another limitation that has already been discussed. The absence of detailed data on symptom severity and comorbidities at the time of patients’ initial presentation in primary care constitutes a methodological limitation that may have influenced clinical decision-making, particularly regarding referrals to the allergy department. Additionally, the exact composition of the Phadiatop mix is not disclosed by the manufacturer; we therefore report allergen groups and representative allergens per European practice, which is sufficient for interpreting atopy screening performance but may not capture minor regional variations.

## Conclusions

To conclude, this study emphasizes the utility of Phadiatop as a first-line screening tool for atopy, particularly in primary care settings, indicating high clinical efficiency in detecting atopy status and the risk of clinical reactions to common inhalant allergens. However, the relatively low referral rate for patients with positive Phadiatop results highlights the need for enhanced education and greater awareness among healthcare professionals to optimize its benefits in managing allergic conditions effectively. Furthermore, this study revealed a diverse range of clinical diagnoses made after referral, with most patients being polysensitized to allergens, confirming the necessity for comprehensive diagnostic procedures following Phadiatop positivity.

## Data Availability

The raw data supporting the conclusions of this article will be made available by the authors, without undue reservation.
